# Factors associated with willingness to use ecological sanitation toilets in Katine sub county Soroti district Uganda: a cross sectional study

**DOI:** 10.1038/s41598-025-20430-x

**Published:** 2025-10-17

**Authors:** Betty Osako Ikiring, David Okia, Charles Okolimong, Jimmy Patrick Alunyo, Richard Katuramu, Annet Tabitha Khainza, David Mukunya, Joseph K. B. Matovu, David Musoke, Peter Olupot-Olupot, Benon Wanume

**Affiliations:** 1 Department of Internal Medicine, Faculty of Health Sciences, Busitema University, Mbale, Uganda; 2 Department of Disease Control and Environmental Health, Makerere University School of Public Health, Kampala, Uganda; 3 Department of Research, Mbale Clinical Research Institute, Mbale, Uganda; 4 Department of Community and Public Health, Busitema University, Mbale, Uganda

**Keywords:** Ecological sanitation, Willingness, Rural sanitation, Uganda, Waste reuse, Health services, Health care, Public health, Epidemiology

## Abstract

Ecological sanitation (EcoSan) toilets provide a sustainable approach to waste management by converting human excreta into usable agricultural inputs. Despite their environmental and health benefits, uptake remains low in many settings. This study investigated the proportion of community members in Katine sub-county, Soroti district, Eastern Uganda, who had ever used EcoSan toilets, their willingness to use them, and the factors associated with this willingness. A cross-sectional survey was conducted among 395 residents using structured questionnaires. Descriptive statistics estimated the proportions of prior use and willingness to use EcoSan toilets. Multivariable logistic regression was used to identify factors independently associated with willingness. Analyses were conducted in Stata 18, and results are presented in tables and figures. Only 18.5% (73/395) of respondents had used EcoSan toilets, and 13.7% (54/395) expressed willingness to use them. In multivariable analysis, key factors negatively associated with willingness included awareness of Ecosan toilets (adjusted odds ratio [AOR] 0.06, 95% CI 0.02–0.16; *p* < 0.0001), willingness to use sanitized fecal matter (AOR 0.06, 95% CI 0.02–0.17; *p* < 0.0001), and perceived ease of use (AOR 0.16, 95% CI 0.05–0.48; *p* = 0.001). Adoption of EcoSan toilets remains low in this setting, primarily due to limited awareness, cultural reservations, and usability concerns. Community sensitisation, culturally appropriate messaging, and user centered toilet design are essential to improving uptake in similar rural environments.

## Introduction

Access to safe sanitation remains one of the most pressing global health challenges. As of 2022, an estimated 2.2 billion people worldwide still lacked safely managed sanitation services, with over 419 million practicing open defecation^1^. The health consequences are severe: inadequate sanitation is a major contributor to the transmission of enteric infections, with diarrheal diseases alone accounting for over 700,000 preventable deaths annually, predominantly among children under five^2^. The United Nations Sustainable Development Goal (SDG) 6 aims to ensure access to adequate and equitable sanitation for all by 2030, emphasizing the urgency of addressing these global disparities^3^.

Sub-Saharan Africa continues to face disproportionately high sanitation deficits. Despite population growth and policy reforms, basic sanitation coverage in the region remains the lowest globally, with only 30% of the population accessing improved sanitation as of 2022^1^. Inadequate sanitation in the region exacerbates cycles of poverty, poor health, and environmental degradation, undermining broader development goals.

Uganda reflects this regional pattern. Although national sanitation initiatives have made incremental progress, challenges persist, particularly in rural settings. According to the Uganda Bureau of Statistics^4^, 22.9% of Ugandans still practice open defecation, while 64% of rural households lack handwashing facilities. These persistent gaps highlight the need for contextually appropriate, sustainable, and user-acceptable solutions that can address both infrastructural and behavioral barriers.

Ecological Sanitation (Ecosan) represents one such approach. Designed to close the loop between sanitation and agriculture, Ecosan technologie, such as Urine-Diverting Dry Toilets (UDDTs), aim to safely manage human excreta while promoting nutrient recycling and environmental protection. Ecosan separates feces and urine at the source, enabling safe treatment and reuse of waste as biofertilizer rich in nitrogen, phosphorus, and potassium^5^. In Uganda, Ecosan was introduced in 1996 and has since been promoted in water-stressed regions and areas with poor soils. Approximately 30,000 units have been installed across households, schools, and public institutions^6^.

Despite its technical and environmental benefits, the widespread adoption of Ecosan remains limited. Several studies across East Africa have documented barriers to acceptance, including concerns about handling human waste, lack of knowledge about the technology, and socio-cultural resistance to using Ecosan by-products in food production^7,9^. In Uganda, empirical studies specifically investigating community willingness to adopt Ecosan remain scarce, with most existing literature focusing on institutional or technical feasibility.

This study addresses that gap by examining the factors associated with household willingness to adopt Ecosan toilets in a rural Ugandan setting. By focusing on Katine sub-county in Soroti district, the research provides updated insights into community-level perceptions, knowledge, and behavioral intentions toward Ecosan use.

## Methodology

### Study design

A cross-sectional study design was conducted using a quantitative method of data collection by administering a structured closed-ended questionnaire to the selected participants. The collected data was generated from factors associated with community willingness to use Ecosan toilets in Katine sub-county, Soroti district.

### Study setting

The study was conducted in Soroti District, located in eastern Uganda at coordinates 1.69° N latitude and 33.62° E longitude. It is part of the broader Teso sub-region, characterized by a high water table and reliance on subsistence farming. The study specifically focused on Katine sub-county, an area selected due to its unique geo-hydrological and physical characteristics, which influence sanitation practices. Katine was also chosen because it was the site of a pilot Ecosan toilet project implemented by AMREF more than a decade ago, providing an opportunity to assess long-term adoption and sustainability.

Soroti District shares borders with Serere to the south, Ngora to the east, Katakwi to the northeast, Amuria to the north, and Lake Kyoga and Kaberamaido to the west. As per the 2014 Uganda National Household Census, Soroti District had a population of 296,833. Katine sub-county, where this study was conducted, consists of eight parishes and 75 villages, with a projected population of 35,072. The area is predominantly occupied by communities engaged in cattle keeping and subsistence agriculture, factors that may influence sanitation choices and the willingness to adopt Ecosan toilets.

### Study population

The study population was drawn from community members in Katine sub-county, Soroti district, where Ecosan toilets had been implemented as part of a pilot project. The target population comprised both users and non-users of Ecosan toilets within this area. For the purpose of this study, the accessible population consisted of heads of households or their representatives aged 18 years and above who were available at the time of data collection. A probability sampling approach was used to select household members from the community to form the sample population for this study.

### Inclusion and exclusion criteria

The study included all adult household members residing in Katine sub-county, Soroti District, regardless of prior use of Ecosan toilets. Individuals who were mentally ill or terminally ill were excluded due to ethical considerations and concerns regarding their ability to provide informed consent or participate meaningfully in the interview process. All participants provided verbal informed consent prior to data collection. 

### Sampling procedure

This study was conducted in all eight parishes of Katine sub county. Two villages were randomly selected from each parish, with 25 respondents chosen from each of the 16 villages. Simple random sampling was used at the village level to minimize selection bias and ensure each individual had an equal chance of selection. This approach enhances sample representativeness and generalizability.

### Sample size

The sample size for the study was determined using Yamane’s formula (1967):

- n = N/ (1 + Nd^2^).
- The researcher used this formula because it is appropriate for the study, given that the average number of household heads of the area, was known, and it provides a reasonable sample size that can be studied within the population.
- n = N/ (1 + Nd^2^)
- where,
- n = sample size representing the minimum number of participants required for statistical validity.
- N = Average number of household heads in Katine
- d = margin of error (5% or 0.05 at 95% confidence level). This allows the probability of making an error in selecting a small representative of the population.
- *Therefore, by substituting*; n = 5745/ (1 + 5745 * 0.05^2^) = 374.5.

After adjusting for the 10% design effect, the final sample size was approximately 413 household heads. However, during data analysis, 18 data points were excluded due to non-responsiveness or incomplete data, leaving a final sample size of 395 with complete records.

### Data collection

Data were collected over a three-week period using a structured questionnaire programmed in KOBO-Toolbox. The tool was selected for its ability to support offline data capture and seamless integration with Stata for statistical analysis. The questionnaire was developed based on a review of existing literature on sanitation adoption, Ecological Sanitation (Ecosan), and behavioral determinants of toilet use in low-resource settings. Items were adapted from validated instruments used in previous studies in East Africa^7,8^ and were reviewed by two independent experts in environmental health and behavioral science to assess content validity.

The tool was translated into Ateso, the predominant local language, and then back-translated into English to ensure semantic and conceptual equivalence. Pre-testing was conducted with 25 respondents in a neighboring sub-county not included in the main study, to assess comprehension, flow, and technical functionality. Minor adjustments were made to phrasing and skip patterns based on feedback. Internal consistency of key multi-item constructs was assessed, with Cronbach’s alpha values ranging from 0.72 to 0.81, indicating acceptable reliability.

Data collection was conducted by three trained research assistants who received a one-day training on study protocols, ethical considerations, and digital data capture. The questionnaire was administered face-to-face to the head of household or another adult representative, randomly selected from eligible households in each village.

### Study variables

Key independent variables included socio-demographic characteristics (e.g., gender, family size, marital status, occupation, education, age, wealth, religion, household income, and house construction materials). These factors were selected based on their potential to influence willingness to adopt Ecosan toilets through their impact on awareness, perceptions, and access. Awareness was defined by whether individuals had heard of Ecosan toilets, and cultural attitudes were assessed by willingness to use Ecosan by-products in agriculture, reflecting acceptance of the technology.

We also examined proximal factors, including community perceptions (attitudes, cultural beliefs, and sanitation behaviors), operation/maintenance factors (knowledge of Ecosan, design features, labor availability, and affordability), and perceived benefits (e.g., agricultural, health, and economic advantages of Ecosan by-products). These were defined through community responses on behaviors like using Ecosan for farming or toilet maintenance.

The dependent variable was willingness to use Ecosan toilets, defined as the degree of acceptance within the community. This was operationalised through individual responses on separating feces and urine and the intent to reuse Ecosan by-products for agriculture.

### Data management

The Researcher thoroughly looked through the questionnaires to ensure completeness. After cleaning, the data were then exported to STATA Ver.18 for analysis.

#### Data analysis

Descriptive statistics were used to summarize respondent characteristics, with categorical variables presented as frequencies and percentages. Associations between independent variables and willingness to use Ecosan toilets were initially assessed using Pearson’s chi-square test at the bivariate level.

Variables with a *p* value < 0.05 in bivariate analysis were included in the multivariable logistic regression model. Additionally, key variables identified a priori based on existing literature, such as age, sex, education, and awareness of Ecosan, were included to adjust for potential confounding. Adjusted odds ratios (AORs) with 95% confidence intervals and corresponding *p *values were reported.

Model specification and goodness-of-fit were assessed using the linktest, which indicated no evidence of model misspecification (*p* > 0.05 for the squared predicted value). Multicollinearity was evaluated using variance inflation factors (VIF), and all included variables showed acceptable VIF values (< 2). Statistical significance was set at *p* < 0.05.

## Results

### Socio-demographic characteristics of the participants

A total of 395 participants were recruited, with a mean age of 38.1 ± 9.3 years. The largest age group consisted of those over 41 years (43.0%), followed by participants aged 31–40 years (27.8%). More than half of the respondents were female (53.7%). In terms of education, the majority had completed primary level education (62.3%), while 13.2% had no formal schooling.

The vast majority of participants (92.2%) were engaged in farming or peasantry. The distribution across wealth quintiles was relatively even, with the largest group being in the poorest category (21.8%). Regarding religion, most respondents identified as Catholic (39.2%) or Protestant (34.4%). The majority of the participants were cohabiting or married (88.1%), and 63.8% of households consisted of 5–10 members (Table 1).

**Table 1:** Participant Social demographics characteristics.

Variables	Frequencies n (395)	Percentages (100.0%)
Age		
< = 30 years	115	(29.1)
31–40 years	110	(27.8)
> 41 years	170	(43.0)
Gender		
Female	212	(53.7)
Male	183	(46.3)
Level of education		
A-LEVEL	7	(1.8)
Certificate	11	(2.8)
Degree/bachelor	2	(0.5)
Diploma	7	(1.8)
Masters	1	(0.3)
No formal schooling	52	(13.2)
O-LEVEL	69	(17.5)
Primary level	246	(62.3)
Occupation		
Business	11	(2.8)
Farming/peasant	364	(92.2)
Government employee	20	(5.1)
Wealth quintiles		
Poorest	86	(21.8)
Poor	70	(17.7)
Middle	82	(20.8)
Rich	79	(20.0)
Richest	78	(19.7)
Religion		
Catholic	155	(39.2)
Protestant	136	(34.4)
Pentecost (born again)	99	(25.1)
Other religions	5	(1.3)
Marital status		
Cohabiting/married	348	(88.1)
Separated/divorced	26	(6.6)
Single	21	(5.3)
Number of people in the household		
< 5	69	(17.5)
5–10 members	252	(63.8)
> 10	74	(18.7)

### Proportion of community use and willingness to adopt Ecosan toilets in Katine sub-county

**Fig. 1:**
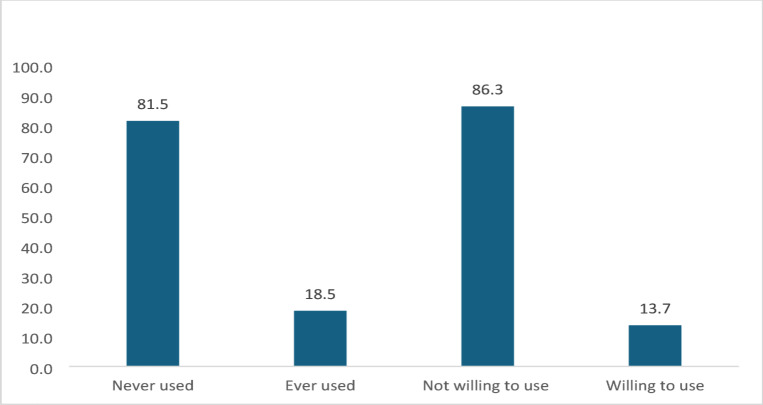
Proportion of community members who have ever used and those who are willing to use Ecosan.

Figure 1 presents the proportions of community members in Katine sub-county who have used and are willing to use Ecosan toilets. A total of 18.5% have used Ecosan toilets, while 81.5% have never used them. Regarding the willingness to adopt Ecosan toilets, 13.7% express interest, while 86.3% are unwilling. See Fig. 1.

### Bivariate results for socio-demographic factors associated with willingness to use Ecosan

Table 2 presents the demographic characteristics of the study participants and their willingness to use Ecosan toilets. Among the age groups, individuals over 41 years exhibited the highest willingness to use Ecosan toilets, with 50.0% indicating willingness compared to 41.9% among those not willing. However, this difference was not statistically significant (*P* = 0.249).

**Table 2:** Bivariate table of socio-demographic factors associated with willingness to use Ecosan.

Variables		Community willingness		*P* value
	All samples = 395 (100%)	Not willing to use = 341 (86.3%)	Willing to use = 54 (13.7%)	
Age				
< = 30 years	115 (29.1)	98 (28.7)	17 (31.5)	0.249
31–40 years	110 (27.8)	100 (29.3)	10 (18.5)	
> 41 years	170 (43.0)	143 (41.9)	27 (50.0)	
Gender				
Female	212 (53.7)	175 (51.3)	37 (68.5)	**0.019**
Male	183 (46.3)	166 (48.7)	17 (31.5)	
Level of education				
A-LEVEL	7 (1.8)	6 (1.8)	1 (1.9)	**0.013**
Certificate	11 (2.8)	11 (3.2)	0 (0.0)	
Degree/bachelor	2 (0.5)	2 (0.6)	0 (0.0)	
Diploma	7 (1.8)	7 (2.1)	0 (0.0)	
Masters	1 (0.3)	0 (0.0)	1 (1.9)	
No formal schooling	52 (13.2)	41 (12.0)	11 (20.4)	
O-LEVEL	69 (17.5)	66 (19.4)	3 (5.6)	
Primary level	246 (62.3)	208 (61.0)	38 (70.4)	
Occupation				
Business	11 (2.8)	9 (2.6)	2 (3.7)	0.473
Farming/peasant	364 (92.2)	313 (91.8)	51 (94.4)	
Government employee	20 (5.1)	19 (5.6)	1 (1.9)	
Wealth quintiles				
Poorest	86 (21.8)	71 (20.8)	15 (27.8)	0.154
Poor	70 (17.7)	59 (17.3)	11 (20.4)	
Middle	82 (20.8)	67 (19.6)	15 (27.8)	
Rich	79 (20.0)	72 (21.1)	7 (13.0)	
Richest	78 (19.7)	72 (21.1)	6 (11.1)	
Religion				
Catholic	155 (39.2)	136 (39.9)	19 (35.2)	0.101
Protestant	136 (34.4)	110 (32.3)	26 (48.1)	
Pentecost (born again)	99 (25.1)	90 (26.4)	9 (16.7)	
Other religions	5 (1.3)	5 (1.5)	0 (0.0)	
Marital status				
Cohabiting/married	348 (88.1)	300 (88.0)	48 (88.9)	0.829
Separated/divorced	26 (6.6)	22 (6.5)	4 (7.4)	
Single	21 (5.3)	19 (5.6)	2 (3.7)	
Number of people in the household				
< 5	69 (17.5)	57 (16.7)	12 (22.2)	**0.009**
5–10 members	252 (63.8)	212 (62.2)	40 (74.1)	
> 10	74 (18.7)	72 (21.1)	2 (3.7)	

Chi-square test used for all comparisons. Bolded *p *values indicate statistical significance at *p* < 0.05.

Gender played a crucial role in willingness, with females showing a significantly higher acceptance at 37/54 (68.5%) compared to only 17/ 54 (31.5%) among males. This association was statistically significant (*P* = 0.019), highlighting the influence of gender on willingness to adopt Ecosan toilets.

The level of education also influenced willingness, particularly among individuals with no formal schooling, where 11/54 (20.4%) expressed willingness compared to 41/341(12.0%) in the same educational category. Notably, among those with primary education, 38/54(70.4%) were willing to use Ecosan toilets, compared to 208/341 (61.0%) of their counterparts not willing. This difference was statistically significant (*P* = 0.013).

The number of people in the household was associated with willingness to use Ecosan. In households with 5–10 members, 40/54 (74.1%) indicated a willingness to use Ecosan toilets, compared to 212/341(62.2%) among those not willing. This finding was statistically significant (*P* = 0.009), suggesting that household size may affect the acceptance of Ecosan toilets. See Table 2.

### Other factors associated with community willingness to use Ecosan

Table 3 displays factors significantly associated with the proportion of community members willing to use Ecosan toilets. Among those willing to use Ecosan toilets, 90.7% had heard of Ecosan, compared to only 24.6% of those not willing, a difference that was statistically significant (*p* < 0.001). In terms of comfort, 100% of individuals willing to use Ecosan toilets expressed comfort, while only 56.0% of those not willing reported the same; also a statistically significant difference (*p* < 0.001).

**Table 3:** Bivariate results of factors associated with the proportion of community members willing to use Ecosan toilets.

Variables		Community willingness		*P* value
	All sample 395 (100.0%)	Not willing to use = 341 (86.3%)	Willing to use = 54 (13.7%)	
Wealth quintiles				
Poorest	86 (21.8)	71 (20.8)	15 (27.8)	0.154
Poor	70 (17.7)	59 (17.3)	11 (20.4)	
Middle	82 (20.8)	67 (19.6)	15 (27.8)	
Rich	79 (20.0)	72 (21.1)	7 (13.0)	
Richest	78 (19.7)	72 (21.1)	6 (11.1)	
Latrine at home				
No	40 (10.1)	35 (10.3)	5 (9.3)	0.82
Yes	355 (89.9)	306 (89.7)	49 (90.7)	
Type of latrine				
Flush toilet	1 (0.3)	1 (0.3)	0 (0.0)	0.36
Local pit latrine	268 (75.5)	226 (73.9)	42 (85.7)	
Pit latrine with cement slab	70 (19.7)	63 (20.6)	7 (14.3)	
Pour flush	1 (0.3)	1 (0.3)	0 (0.0)	
VIP latrine	15 (4.2)	15 (4.9)	0 (0.0)	
Heard of ECOSAN				
No	133 (33.7)	84 (24.6)	49 (90.7)	** < 0.001**
Yes	262 (66.3)	257 (75.4)	5 (9.3)	
Used ECOSAN				
No	322 (81.5)	268 (78.6)	54 (100.0)	** < 0.001**
Yes	73 (18.5)	73 (21.4)	0 (0.0)	
Comfortable using ECOSAN				
No	96 (72.2)	47 (56.0)	49 (100.0)	** < 0.001**
Yes	37 (27.8)	37 (44.0)	0 (0.0)	
Willing to eat from ECOSAN gardens				
No	60 (15.2)	16 (4.7)	44 (81.5)	** < 0.001**
Yes	335 (84.8)	325 (95.3)	10 (18.5)	
ECOSAN cheaper to construct				
No	376 (95.2)	322 (94.4)	54 (100.0)	0.075
Yes	19 (4.8)	19 (5.6)	0 (0.0)	
Cultural leaders support ECOSAN				
No	121 (30.6)	85 (24.9)	36 (66.7)	** < 0.001**
Yes	274 (69.4)	256 (75.1)	18 (33.3)	
Religious support for ECOSAN				
I don’t know	209 (52.9)	158 (46.3)	51 (94.4)	** < 0.001**
No	31 (7.8)	30 (8.8)	1 (1.9)	
Yes	155 (39.2)	153 (44.9)	2 (3.7)	
Use ECOSAN people with disabilities				
No	165 (41.8)	116 (34.0)	49 (90.7)	** < 0.001**
Yes	230 (58.2)	225 (66.0)	5 (9.3)	
Knowledgeable about ECOSAN				
No	306 (77.5)	256 (75.1)	50 (92.6)	**0.004**
Yes	89 (22.5)	85 (24.9)	4 (7.4)	
Children (0–5) know how to use ECOSAN				
No	375 (94.9)	321 (94.1)	54 (100.0)	0.068
Yes	20 (5.1)	20 (5.9)	0 (0.0)	
Use sanitized fecal matter by community members				
No	207 (52.4)	157 (46.0)	50 (92.6)	** < 0.001**
Yes	188 (47.6)	184 (54.0)	4 (7.4)	
Community members willing to eat ECOSAN-grown food				
No	89 (22.5)	49 (14.4)	40 (74.1)	** < 0.001**
Yes	306 (77.5)	292 (85.6)	14 (25.9)	
Willing to clean ECOSAN toilet				
No	151 (38.2)	98 (28.7)	53 (98.1)	** < 0.001**
Yes	244 (61.8)	243 (71.3)	1 (1.9)	
Willing to empty ECOSAN toilet				
No	188 (47.6)	135 (39.6)	53 (98.1)	** < 0.001**
Yes	207 (52.4)	206 (60.4)	1 (1.9)	
Who should clean ECOSAN toilet				
Children	4 (1.0)	4 (1.2)	0 (0.0)	** < 0.001**
Everybody	64 (16.2)	64 (18.8)	0 (0.0)	
Men	134 (33.9)	131 (38.4)	3 (5.6)	
We call someone	184 (46.6)	133 (39.0)	51 (94.4)	
Women	9 (2.3)	9 (2.6)	0 (0.0)	
Ease of using ECOSAN				
Difficult	176 (44.6)	126 (37.0)	50 (92.6)	** < 0.001**
Easy	150 (38.0)	149 (43.7)	1 (1.9)	
Moderate	51 (12.9)	48 (14.1)	3 (5.6)	
Very difficult	5 (1.3)	5 (1.5)	0 (0.0)	
Very easy	13 (3.3)	13 (3.8)	0 (0.0)	
Teachers/pupils willing to use ECOSAN				
I don’t know	300 (75.9)	247 (72.4)	53 (98.1)	** < 0.001**
No	3 (0.8)	2 (0.6)	1 (1.9)	
Yes	92 (23.3)	92 (27.0)	0 (0.0)	
Health benefits from ECOSAN				
I don’t know	250 (63.3)	197 (57.8)	53 (98.1)	** < 0.001**
No	30 (7.6)	29 (8.5)	1 (1.9)	
Yes	115 (29.1)	115 (33.7)	0 (0.0)	
Willing to install ECOSAN at home				
Not willing	48 (12.2)	36 (10.6)	12 (22.2)	**0.001**
Reluctant	281 (71.1)	239 (70.1)	42 (77.8)	
Very willing	1 (0.3)	1 (0.3)	0 (0.0)	
Willing	65 (16.5)	65 (19.1)	0 (0.0)	
Willing to reuse urine/faeces				
Not willing	6 (1.5)	6 (1.8)	0 (0.0)	** < 0.001**
Reluctant	140 (35.4)	91 (26.7)	49 (90.7)	
Very willing	10 (2.5)	10 (2.9)	0 (0.0)	
Willing	239 (60.5)	234 (68.6)	5 (9.3)	

Chi-square test used for all comparisons. Bolded *p* values indicate statistical significance at *p* < 0.05*.*

A significant difference was observed regarding the willingness to eat food grown in gardens where Ecosan by-products are used: 81.5% of those willing to use Ecosan indicated readiness to eat from such gardens, compared to only 4.7% of those not willing (*p* < 0.001). Support from cultural leaders was notably higher among those willing to adopt Ecosan, with 66.7% reporting such support, versus 24.9% of those not willing (*p* < 0.001).

Among those willing, 92.6% reported being knowledgeable about Ecosan, compared to 75.1% of those not willing, with a statistically significant difference (*p* = 0.004). Willingness to clean Ecosan toilets was reported by 98.1% of the willing group, compared to 28.7% of the not willing group (*p* < 0.001). Similarly, 98.1% of those willing indicated they would empty Ecosan toilets, while only 39.6% of those not willing expressed the same willingness (*p* < 0.001). See Table 3.

### Multivariate results for factors associated with community willingness to use Ecosan toilets

In multivariable analysis, awareness of Ecosan toilets was associated with significantly lower odds of willingness to use Ecosan toilets (AOR 0.06, 95% CI 0.02–0.16; *p* < 0.0001). Similarly, respondents willing to use sanitized fecal matter (AOR 0.06, 95% CI 0.02–0.17; *p* < 0.0001) and those who perceived Ecosan toilets as easy to use (AOR 0.16, 95% CI 0.05–0.48; *p* = 0.001) also had reduced odds of willingness,. see Table 4.

**Table 4:** Multivariate results for factors associated with community willingness to use Ecosan.

Variable	COR (95% CI)	*p *value	AOR (95% CI)	*p *value
Ever heard of ECOSAN toilet				
No	1		1	** < 0.0001**
Yes	0.39(0.29 0.51)	< 0.0001	0.06(0.02,0.16)	
Community members willingly using sanitize fecal matters				
No	1		1	** < 0.0001**
Yes	0.35(0.25, 0.49)	< 0.0001	0.06(0.02, 01.7)	
Level of use of ECOSAN toilet				
Difficult	1		1	
Very difficult	5.25(0.52,52.5)	0.158	0.10(0.00, 2.23)	0.148
Easy	9.37(4.34,20.2)	< 0.0001	0.16(0.05,0.48)	**0.001**
Very easy	3.81(0.72,20.1)	0.115	0.17(0.01,3.02)	0.267

Goodness-of-fit test (Prob > chi^2^ = 0.0000): linktest[_hatsq (*p *value = 0.89)]. Significant values are in bold.

## Discussion

This study is among the first in Uganda to comprehensively assess factors influencing willingness to adopt Ecosan toilets, providing valuable insights for sanitation policy and intervention strategies. Despite the known benefits of Ecosan technologies, such as environmental sustainability and resource efficiency, adoption remains low in Soroti District. Only 18.5% of community members reported having ever used Ecosan toilets, and just 13.7% expressed willingness to use them, indicating limited penetration of the technology in this population.

The low usage and willingness to adopt Ecosan toilets may be influenced by limited awareness, cultural preferences for traditional sanitation methods, and practical barriers such as cost and maintenance concerns. Misconceptions about hygiene and unease with handling composted waste further limit acceptance. These findings align with evidence from Malawi, where few property owners intended to adopt Ecosan due to expense, complexity, and compatibility issues, with only around 16% expressing any interest even when pit latrines were problematic^10^. In Rwanda, studies found that only 39.4% of households were better users of Ecosan, which is still low the same as what we found in Soroti^11^. Together, these studies highlight how awareness and financial and practical constraints strongly shape adoption.

Our study identified that knowledge of Ecosan toilets, acceptance of sanitized fecal matter, and perceived ease of use are important factors influencing willingness to adopt. Awareness plays a crucial role by potentially increasing understanding of the benefits of Ecosan technologies, which may be shared through sanitation workshops and community engagement. However, acceptance of sanitized fecal matter remains a significant cultural barrier, as strong taboos and skepticism about human waste reuse persist in the community. Similar cultural challenges have been documented in Rwanda, where excreta-related taboos limited Ecosan uptake^11^. In contrast, findings from Burkina Faso suggest that training emphasizing agricultural reuse can improve acceptance, highlighting the importance of tailoring interventions to local cultural contexts^12^.

Perceived ease of use also influences willingness, underscoring the need for sanitation technologies that are convenient and user-friendly. This is consistent with findings from South Africa, where user acceptability strongly affected sanitation technology adoption^13^. Overall, these results suggest that increasing Ecosan uptake requires addressing both cultural attitudes and practical usability.

Comparisons with studies from Malawi, Kenya, and Burkina Faso emphasize the importance of contextual factors such as subsidy levels, cultural norms, and implementation strategies. The relatively lower willingness in our study may reflect fewer subsidies, less community involvement, or stronger cultural resistance compared to other settings. Deeper examination of these factors is needed to inform effective program design.

### Strengths and limitations

This study provides important, locally relevant insights into Ecosan adoption willingness in Eastern Uganda. The community-centered approach captures perceptions critical for tailoring future sanitation interventions. However, the cross-sectional design limits the ability to infer causality, and reliance on self-reported data introduces potential bias. Selection bias from nonresponse and exclusion of vulnerable groups may also affect generalizability. Longitudinal and qualitative studies are recommended to explore changes in adoption over time and deeper cultural barriers.

### Recommendations

Interventions should go beyond broad education and generic workshops by implementing evidence-based, context-specific approaches. Examples include community demonstration plots to showcase safe reuse of sanitized excreta, microfinance schemes to reduce cost barriers, and active engagement of community leaders to foster ownership and trust. Design improvements to enhance the user-friendliness and maintenance of Ecosan toilets are also essential to overcoming practical challenges.

## Conclusion

In this rural Ugandan setting, adoption of Ecosan toilets remains limited. Willingness to use Ecosan is influenced by awareness, cultural acceptance of sanitized waste, and perceptions of ease of use. These findings highlight the need for targeted, culturally sensitive interventions and suggest the importance of rigorous evaluation through longitudinal or intervention studies before scaling up policies.

## Data Availability

The datasets used and/or analyzed during the current study are available from the corresponding author upon reasonable request.
